# Positive regulatory role of sound vibration treatment in *Arabidopsis thaliana* against *Botrytis cinerea* infection

**DOI:** 10.1038/s41598-017-02556-9

**Published:** 2017-05-30

**Authors:** Bosung Choi, Ritesh Ghosh, Mayank Anand Gururani, Gnanendra Shanmugam, Junhyun Jeon, Jonggeun Kim, Soo-Chul Park, Mi-Jeong Jeong, Kyung-Hwan Han, Dong-Won Bae, Hanhong Bae

**Affiliations:** 1 Department of Biotechnology, Yeungnam University, Gyeongsan, Gyeongbuk 38541 Republic of Korea; 2 Department of Biology, College of Science, United Arab Emirates University, Al Ain, 15551 United Arab Emirates; 3 National Institute of Agricultural Sciences, Rural Development Administration, Wanju, Jeollabuk 55365 Republic of Korea; 4 Department of Horticulture and Department of Forestry, Michigan State University, East Lansing, MI 48824-1222 USA; 5 Central Instrument Facility, Gyeongsang National University, Jinju, Gyeongnam 52828 Republic of Korea

## Abstract

Sound vibration (SV), a mechanical stimulus, can trigger various molecular and physiological changes in plants like gene expression, hormonal modulation, induced antioxidant activity and calcium spiking. It also alters the seed germination and growth of plants. In this study, we investigated the effects of SV on the resistance of *Arabidopsis thaliana* against *Botrytis cinerea* infection. The microarray analysis was performed on infected Arabidopsis plants pre-exposed to SV of 1000 Hertz with 100 decibels. Broadly, the transcriptomic analysis revealed up-regulation of several defense and SA-responsive and/or signaling genes. Quantitative real-time PCR (qRT-PCR) analysis of selected genes also validated the induction of SA-mediated response in the infected Arabidopsis plants pre-exposed to SV. Corroboratively, hormonal analysis identified the increased concentration of salicylic acid (SA) in the SV-treated plants after pathogen inoculation. In contrast, jasmonic acid (JA) level in the SV-treated plants remained stable but lower than control plants during the infection. Based on these findings, we propose that SV treatment invigorates the plant defense system by regulating the SA-mediated priming effect, consequently promoting the SV-induced resistance in Arabidopsis against *B*. *cinerea*.

## Introduction

Plants have evolved themselves against environmental challenges with effective physiological and developmental modifications. For instance, plants undergo significant changes in their growth and development in response to mechanical stimuli (e.g., touch and wind), which is called thigmomorphogenesis^1^. It is a complex plant response that involves changes in growth, flowering time, senescence, pithiness, chlorophyll content, hormone level, biotic and abiotic stress resistance, and control of stomatal aperture^1^. Interestingly, recent studies have indicated that sound vibration (SV) may act as a pressure wave, triggering thigmomorphogenesis (reviewed in^2^). The available evidence indicates that plants are responsive towards natural sounds, thus suggesting an ecological and environmental relevance of plant-acoustic interaction. For instance, the ‘Buzz Pollinated’ plants release pollens from anthers when exposed to a specific frequency of sound produced by bee buzz^3^. A large number of plant species are known to possess such peculiar floral behavior^3^. It was identified that pretreatment with vibrations caused by chewing sound of caterpillar can induce the Arabidopsis chemical defense^4^. However, leafhopper song failed to prime the defense response in the same study. This finding suggests that plants evolved appreciably enough to selectively respond to particular SV. Moreover, the elevated levels of polyamines and oxygen uptake in Chinese cabbage after the exposure of green music (e.g. certain natural music like bird’s singing, cricket’s stridulating etc.) also substantiate the ecological significance^5^. Additionally, several other plausible ecological relevance of plant-acoustic interaction has been discussed by Mishra *et al*.^2^. These examples strongly suggest that plants have evolved sensitivity towards ecologically relevant SV, although the molecular mechanism is poorly understood.

The beneficial effects of synthetic SV on plant growth and development were studied in various plant species, including paddy, wheat, tomato, lettuce, spinach, cucumber and strawberry^6^. These beneficial effects include stimulation of seed germination and an increase in the number of fruits, plant height, tiller growth and crop yield. Furthermore, SV exposure with 1000 Hertz (Hz) caused various biochemical changes in *Chrysanthemum*, *Dendrobium*, and *Actinidia* such as induction of soluble sugar content, protein, antioxidant enzyme activity and optimized energy metabolism^2, 6^. Moreover, *Chrysanthemum* exposed to 1400 Hz SV showed accumulation of indole-3-acetic acid (IAA) and reduction of abscisic acid (ABA)^7^. Young roots of maize were grown toward acoustic emissions (220 Hz), showing thigmotropism or sound tropism^8^. These aforementioned studies reveal the beneficial effects of SV on plant, suggesting the existence of sophisticated molecular mechanisms for SV perception and signal transduction without any specialized sensory organs like ears. It is evident that external auditory organs are not obvious for SV perception in kingdom Animalia^8^. Nonetheless, how plants perceive SV remains elusive. It has been hypothesized that the cytoskeleton-plasma membrane-cell wall interface has an important role in SV perception^9^. Moreover, the expression analysis of SV-regulated genes after touch treatment hints at the possibility that SV is perceived as a stimulus distinct from touch, even though there is close resemblance between these two stimuli at molecular level^10^.

Further, SV can result in some molecular alterations that may enable plants to cope up with future stresses. Few reports suggested that SV also acts as a priming agent like other mechanical stimuli. For example, SV could induce disease resistance in strawberry^11^. Similarly, Arabidopsis was reported to perceive SV generated from insect herbivore and subsequently elicit systematic chemical defense^4^. Even pre-exposure of SV could increase drought stress tolerance in rice^12^. It is suggested that plant cells have the ability to get gradually primed when exposed to certain environmental or chemical challenges^13^. According to the priming effect hypothesis, repeated external stimulation can be imprinted as molecular-memory in the form of epigenetic marks or protein synthesis within plant cells, which then prepare the plant for sturdy response against future biotic or abiotic stresses^14^. This priming effect may explain how previous exposure to mild stress enables the plant to respond effectively to new stress factors. Such priming can be achieved by mechanical stimulation as well^15^. For instance, daily repetitive or dose dependent touch treatment on Arabidopsis leaves increased resistance against *Botrytis cinerea* ^16, 17^.

Considering that SV treatment might be an easy and inexpensive means to increase stress resistance in crops, herein, we investigated the effect of SV on biotic stress response. A better understanding of the SV-mediated priming mechanisms might have direct implications on the agriculture industry. As a step toward unraveling the mechanisms by which plants respond to SV and increase their resistance against biotic stresses, we investigated SV-mediated transcripts and hormonal changes during fungal infection. In this report, we described that SV pre-treatment plays a positive role in Arabidopsis defense against *B*. *cinerea*.

## Results

### SV treatment with 1000 Hz increases disease resistance against *B*. *cinerea* in *A*. *thaliana*

**Figure 1:**
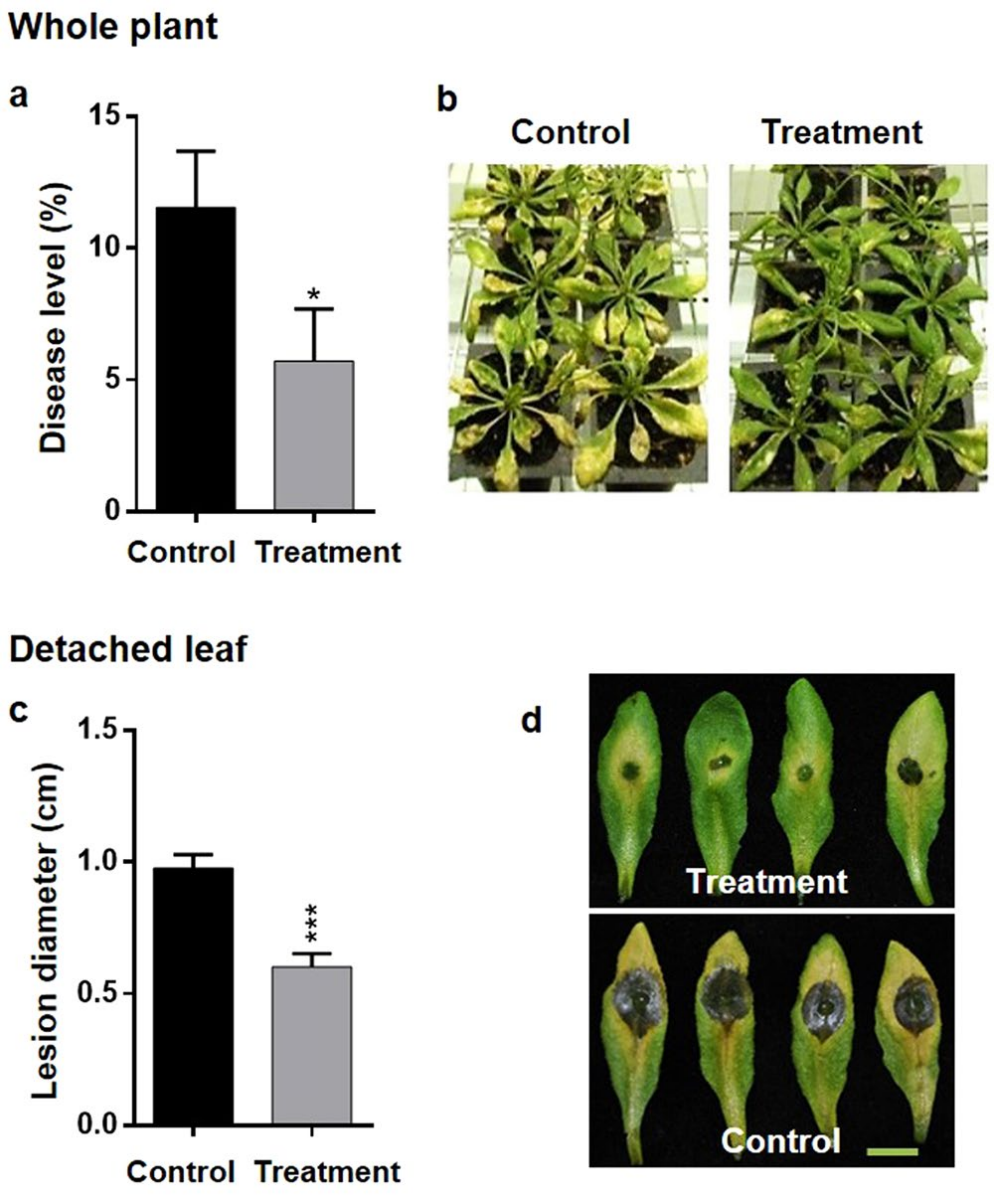
Reduction of disease symptoms after infection with *B*. *cinerea* in *A*.*thaliana* exposed to 1000 Hz sound vibration. **(a)** Percentage of disease level in whole plant assay. Percentage of disease level was calculated by counting fully senescent leaves at 72 h post inoculation (hpi). Bars represent mean and standard error of four biological replications. **(b)** The disease phenotype of whole plants at 72 hpi. Plants exposed to sound vibration (daily 3 h for 10 days, treatment) and control were inoculated with fungal spores and kept under growth room condition with high humidity. **(c)** Lesion diameter in detached leaf assay. Lesion diameter was measured at 72 hpi. Bars indicate the means of the lesion diameter with standard errors in 40 detached leaves. **(d)** The lesion phenotype of detached leaves. Detached leaves exposed to a 1000 Hz sound vibration (upper) and control (lower) at 72 hpi of *B*. *cinerea* spores. bar = 1 cm. *P*-value ranges are marked by asterisks: ^***^ *P* < 0.01, ^**^ 0.01 < *P* < 0.05, ^*^ *P* < 0.1.

In the preliminary study, three different frequencies [500, 1000, and 3000 Hz] with constant amplitude [100 decibels (dB)] were separately applied (daily 3 h for 10 days) in Arabidopsis for the induction of disease resistance against *B*. *cinerea* (Supplementary Figs S1 and S2). Pre-treatment with 1000 Hz showed significant increase in resistance against *B*. *cinerea* (Fig. 1a). In the whole plant assay, significant difference in disease level was observed between control and treatment at 72 h post infection (hpi) (Fig. 1b). In the detached leaf assay, lesion diameter and disease progression were observed at 72 hpi (Fig. 1c and d). Detached leaf assay also confirmed the findings of the whole plant assay, showing that SV treatment increased disease resistance. Since 1000 Hz with 100 dB treatment induced the highest disease resistance against *B*. *cinerea* in both whole plants and detached leaves, we chose 1000 Hz for further study.

### SV treatment with 1000 Hz alters transcriptomic profiles in *A*. *thaliana* during *B*. *cinerea* infection

As SV pre-treatment increased the disease resistance of Arabidopsis against *B*. *cinerea*, we investigated the transcriptome changes using Affymetrix microarray. Samples from three different time points were analyzed through microarray: (1) right after the 10^th^ day of 3 h SV treatment (0 h time point) to analyze the effect of SV exposure on gene expression, (2) after *Botrytis* spore inoculation (12 and 24 hpi time points) to compare the defense-related gene expression between SV-treated and control plants. A schematic view of SV treatment method, pathogen inoculation and sample harvesting time is shown in Supplementary Fig. S1.

**Figure 2:**
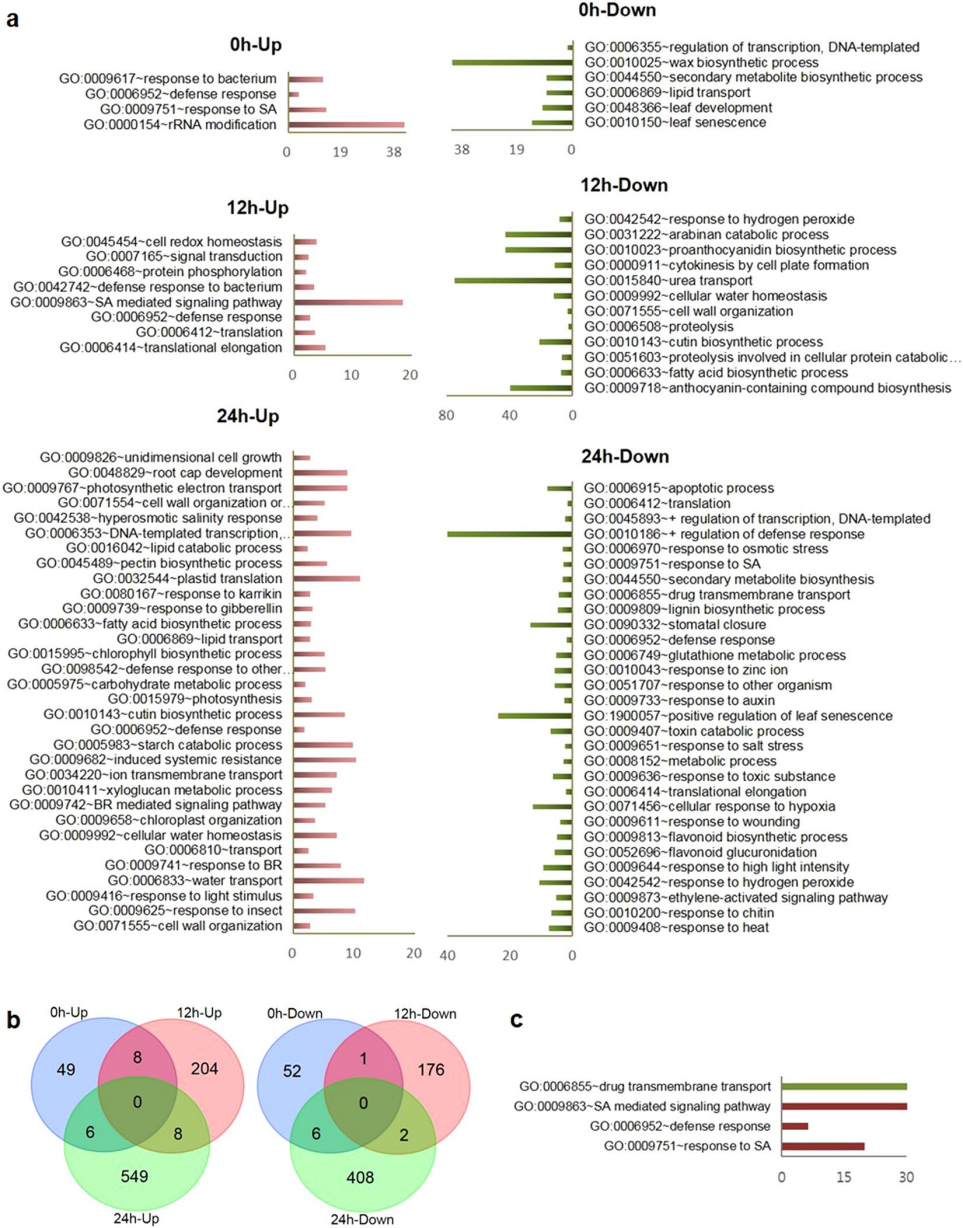
Gene ontology enrichment analysis of the differentially expressed genes after infection with *B*. *cinerea* in *A*. *thaliana* exposed to 1000 Hz sound vibration. (**a**) The differentially expressed genes (*P* < 0.05, fold change > 1.5) were categorized according to gene ontology (GO) biological process. The detailed analyses of all GO classes are available in Supplementary Table S1. (**b**) Venn diagram represents the number of overlapping genes between the analyzed time points. The time (0, 12, and 24 h) indicates h post inoculation (hpi). Up and down indicate the up- and down- regulated genes respectively at given time points. (**c**) Enriched GO of biological process within the list of genes found to be common at least in two different time points. Red and green color bars indicate up- and down- regulated genes, respectively. GO enrichment analysis was performed through DAVID bioinformatics resources at *P* < 0.05. Microarray analysis was performed with three biological replications.

**Figure 3:**
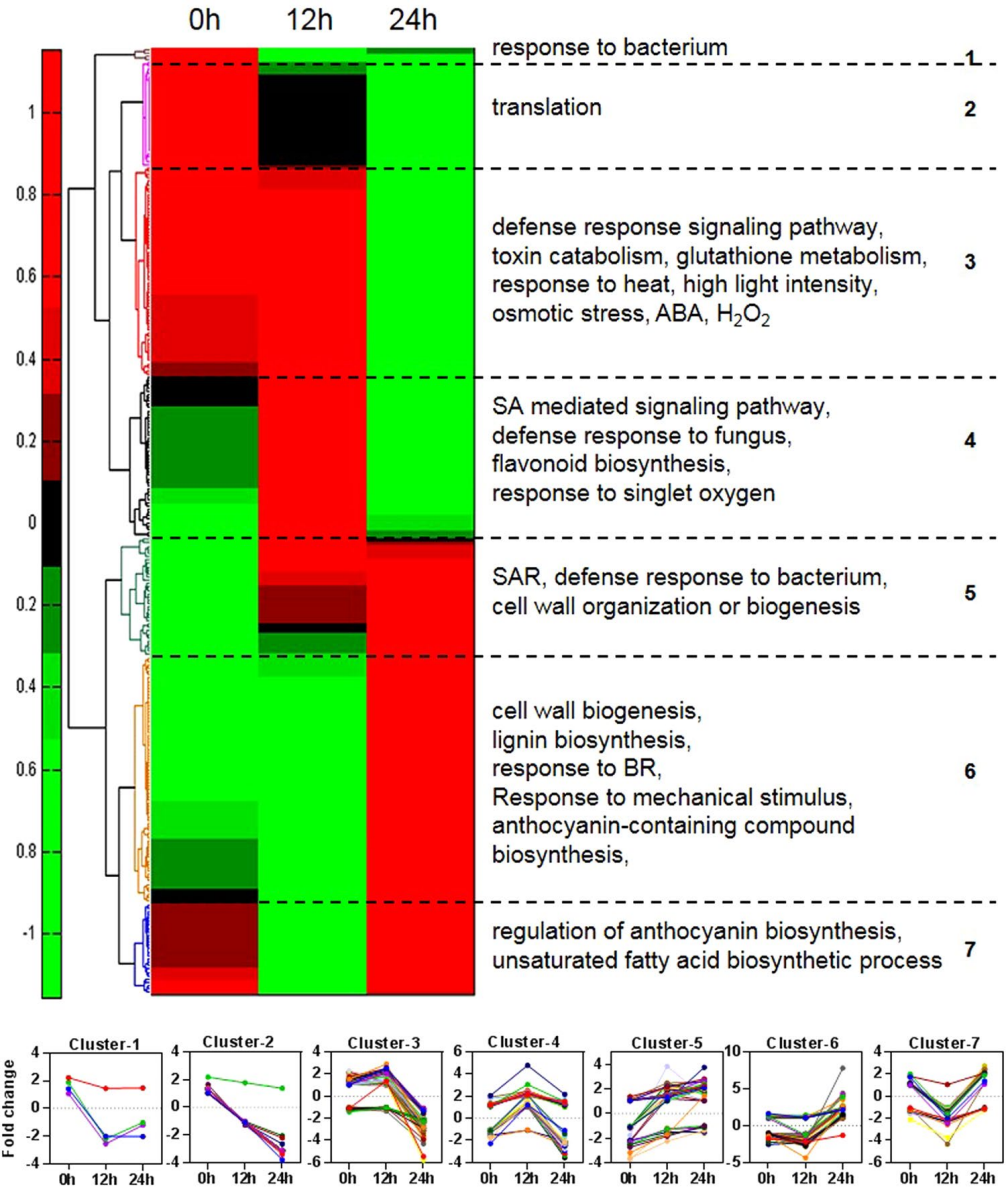
Hierarchical clustering of differentially expressed genes after infection with *B*. *cinerea* in *A*. *thaliana* exposed to 1000 Hz sound vibration. A total of 280 differentially expressed (2 fold as threshold) genes were classified in seven clusters (1 to 7) through hierarchical clustering with Z-score normalization. Red and green colors indicate up- and down- regulated genes, respectively. Enriched gene ontology of biological process (*P* < 0.05) were mentioned in every cluster. Details of the genes and enrichment analysis of all GO classes are available in Supplementary Table S2. Line graphs at bottom indicate the fold change of the genes of each cluster. The time (0, 12, and 24 h) indicates h post inoculation (hpi).

The largest number of differentially expressed genes was observed at 24 hpi between control and SV-treated plants. The genes that were differentially expressed (*P* < 0.05 and fold change >1.5) were subjected to the gene ontology (GO) analysis through DAVID bioinformatics resources. List of these genes and details of the enrichment analysis (*P* < 0.05) of 3 GO classes (biological process, molecular function, and cellular component) are available in Supplementary Table S1. Enrichment analysis of biological process indicates that several defense-related and SA-responsive/signaling genes were up-regulated at 0 and 12 hpi in the SV-treated plants compared to control plants (Fig. 2a). Furthermore, the genes related to defense response, induced systemic resistance and cell wall organization were notably up-regulated at 24 hpi in the SV-treated plants. Interestingly, several genes related to abiotic stress response (like- osmotic, salt, high light intensity and heat) were down-regulated in the SV-treated plants after 24 hpi compared to control plants (Fig. 2a). The Venn diagrams indicate that not even a single gene was commonly up- or down- regulated at three different time points (Fig. 2b). However, there were few up- and down- regulated genes which were common between the two different time points (0 vs 12, 0 vs 24, and 12 vs 24 hpi). Totally, 22 up-regulated and 9 down-regulated genes were noted to be common while considering their expressions at least at two different time points, which were further analyzed through GO enrichment study (*P* < 0.05). Here too, the up-regulated common genes were enriched with SA responsive/signaling GO term (Fig. 2c). At 0, 12 and 24 hpi, a total of 7, 35 and 93 genes were up-regulated (fold change > 2) compared to control plants, respectively; while 14, 34 and 112 genes were down-regulated (fold change > 2), respectively (Supplementary Table S2). A total of 280 differentially expressed (2 fold as threshold) genes were hierarchically clustered with Z-score normalization. Seven clusters were identified based on the expression kinetics of these genes (Fig. 3). Simultaneously, GO enrichment study of each cluster was performed at *P* < 0.05. Details of the enrichment analysis of 3 GO classes are available in Supplementary Table S2. Defense-responsive and SA signaling pathway genes mainly belong to the cluster 3 and 4 (Fig. 3). Broadly, the genes of these two clusters were up-regulated at 12 hpi in the SV-treated plants compared to control plants. Systemic acquired resistance (SAR) and cell wall organization /biogenesis related genes belong to clusters 5 and 6 where the genes were broadly up-regulated at 24 hpi in the SV-treated plants (Fig. 3).

### Validation by qRT-PCR

**Figure 4:**
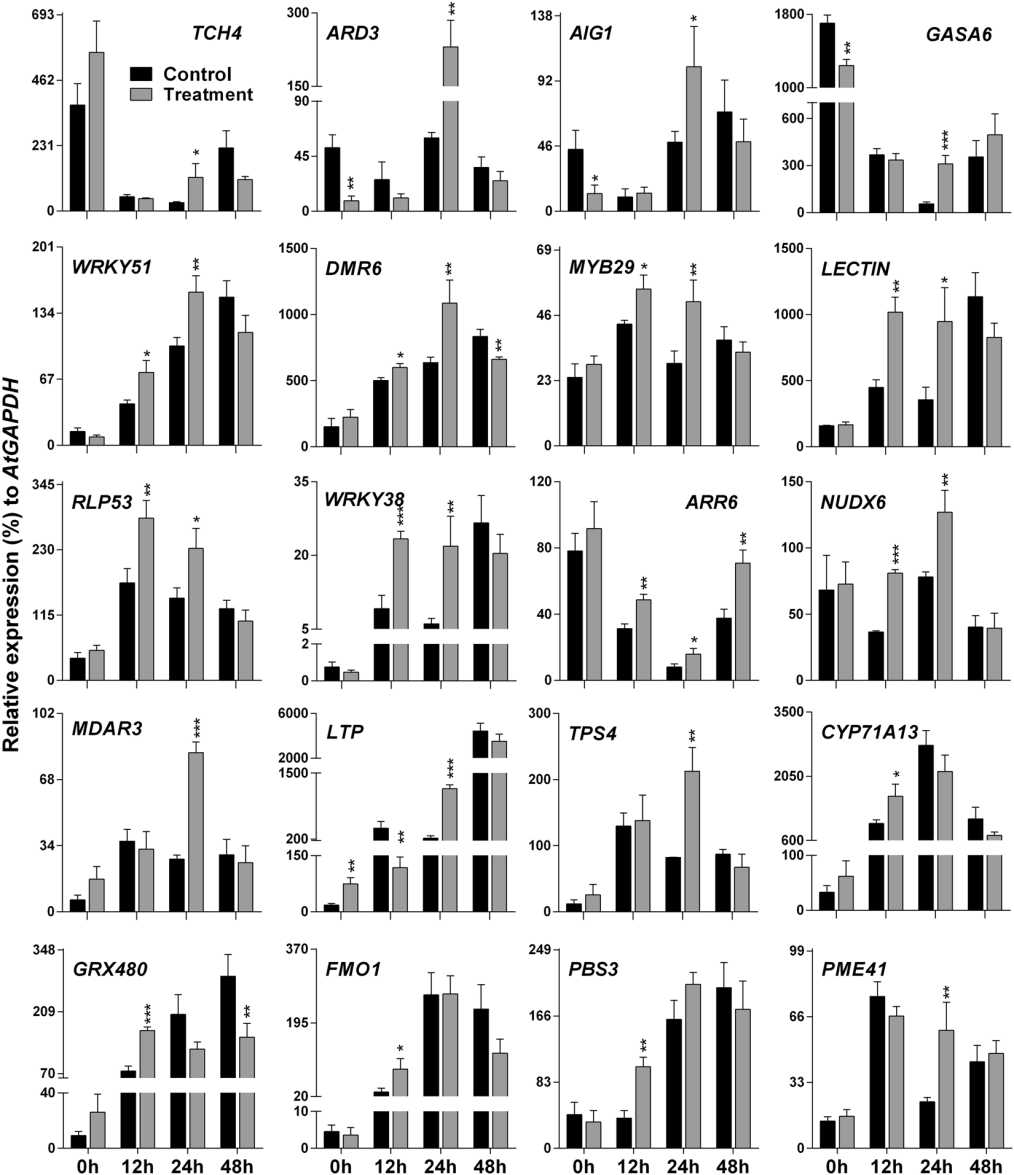
Validation of microarray data using qRT-PCR. Microarray results of twenty representative genes are confirmed by qRT-PCR. Expression of each gene in the *A*. *thaliana* exposed to a 1000 Hz sound vibration (gray) was compared with that of control plants (black). *AtGAPDH* was used for normalization. Bars represent mean and standard error of three biological replications. The time (0, 12, 24 and 48 h) indicates h post inoculation (hpi). Details of genes and their fold changes in microarray experiment are available in Table 1. *P-*value ranges are marked by asterisks: ^***^ *P* < 0.01, ^**^ 0.01 < *P* < 0.05, ^*^ *P* < 0.1.

Microarray results were validated using qRT-PCR in whole plant system (Fig. 4). A total of twenty genes (fold change >2) involved in disease resistance were selected for qRT-PCR confirmation. For a quick reference, the fold change based on microarray results and functions of these genes are mentioned in Table 1. *TCH4*, a touch-inducible gene, was induced in the SV-treated plants compared to control plants at 0 and 24 hpi. Three genes (*ARD3*, *AIG1*, and *GASA6*) were strongly up-regulated at 24 hpi, although they were down-regulated at 0 hpi; while seven genes (*WRKY51*, *DMR6*, *MYB29*, *LECTIN*, *RLP53*, *WRKY38* and *NUDX6*) were up-regulated at 12 and 24 hpi in the SV-treated plants. AIG1 is involved in recognition of bacterial pathogens carrying *avrRpt2*, the avirulence gene^18^. Expression of *DMR6* can be induced by SAR and benzothiadiazole (BTH)^19^. *LECTIN* has vital role in plant defense against pathogens and predators^20^. Moreover, lectin-like protein (SAI-LLP1) in Arabidopsis is involved in SA-mediated immunity against *Pseudomonas syringae* ^21^. *RLP53* encodes a member of receptor-like proteins (RLPs) which are involved in early pathogen recognition and activation of protective immune signaling in plants^22^. *NUDX6* has significant impact on plant immune response as a positive regulator of NPR1-dependent SA signaling pathways^23^. Both WRKY51 and WRKY38 seem to have a potential role in SA-mediated downstream signaling^24^. MYB29 is involved in the biosynthesis of aliphatic glucosinolate, a sulphur containing defense molecules^25^. *ARR6*, an important member of cytokinin (CK) signaling network, showed higher level in the SV-treated plants than control at all-time points after fungal inoculation. *MDAR3*, *LTP*, and *TPS4*, were up-regulated in the SV-treated plants at 0 and 24 hpi. *LTP* is a bacterial flagellin (flg22)-induced gene^26^. *CYP71A13*, *GRX480*, *FMO1*, and *PBS3* were up-regulated at 12 hpi, while *PME41* was strongly up-regulated at 24 hpi in the SV-treated plants compared to control. *PME41* synthesizes a member of pectin methylesterases that has an important role in active immune response^27^. *PBS3* (also known as GH3.12) is important for pathogen-induced SA accumulation, and directly regulates the synthesis of important upstream molecule of SA^28^. *FMO1*, has a crucial role in basal resistance to invasive virulent pathogens and functions as a positive regulator of EDS1, an important player in SA signaling^29^. *GRX480* is SA-inducible and requires NPR1 (also known as NIM1 or SAI1)^30^. On the basis of our microarray and qRT-PCR data, the overall defense mechanism in SV-treated Arabidopsis appears to be effectively triggered at early stage (12 and 24 hpi) of infection and was mediated by SA signaling.

**Table 1:** List of selected genes which are involved in defense and phytohormone signaling.

TAIR ID	Gene	Function	Fold change		
			0 h	12 h	24 h
At5g57560	Xyloglucan endotransglucosylase (TCH4)	Cell wall biogenesis			2.3**
At2g26400	Acireductone dioxygenase 3 (ARD3)	Methionine metabolism	2	4.7	2.1
At1g33960	avrrpt2-induced gene 1 (AIG1)	Response to bacterium		2.4	1.7
At1g74670	GA-stimulated Arabidopsis 6 (GASA6)	GA signaling			3.7***
At5g64810	WRKY protein 51 (WRKY51)	Defense, SA		2.4***	
At5g24530	Downy mildew resistant 6 (DMR6)	Flavonoid biosynthesis, SA	2.2**	1.4	1.4
At5g07690	MYB domain protein 29 (MYB29)	Defense response		1.4	2.1**
At5g03350	Lectin	SAR, SA		2.1**	2.8***
At5g27060	Receptor like protein 53 (RLP53)	Signaling, Defense		2.4***	2.5***
At5g22570	WRKY protein 38 (WRKY38)	Transcription, SA		1.8***	2***
At5g62920	Response regulator 6 (ARR6)	CK-activated signaling			2.4***
At2g04450	Nudix hydrolase homolog 6(NUDX6)	SA-mediated signaling	1.9	2.4**	
At3g09940	Monodehydroascorbate reductase (MDAR3)	Oxidation-reduction process		2.1*	1.5
At4g12500	Lipid-transfer protein (LTP)	Lipid transport			3.9**
At1g61120	Terpene synthase 4 (TPS4)	Diterpenoid biosynthesis		2.4	
At2g30770	Cytochrome P450 (CYP71A13)	Camalexin biosynthesis	2		
At1g28480	Glutaredoxin-GR480 (GRX480)	Cell redox homeostasis, SA	1.7**	2***	
At1g19250	Flavin-dependent monooxygenase (FMO1)	Defense	1.7	2.5	
At5g13320	GH3-like defense gene (PBS3)	Defense, SA		2.2*	
At4g02330	Pectin methylesterase 41 (PME41)	Pectin metabolism			2.2***
At4g12470	Azelaic acid induced 1 (AZI1)	Defense, SAR			2.0*
At5g36970	NDR1/HIN1-like 25 (NHL25)	Defense, SA		2.1***	
At1g02450	NIM1-interacting 1 (NIMIN1)	Defense, SAR, SA		2.1***	
At2g14610	Pathogenesis-related protein 1 (PR1)	Defense, SAR, SA	−1.7	3.8	
At1g66100	Pathogenesis-related protein (PR)	Defense			2.2***
At5g24780	Vegetative storage protein 1 (VSP1)	Defense, JA	−1.8	-2.2	7.8***

Numbers indicate the fold changes observed through microarray experiment. Genes are responsive to salicylic acid (SA), jasmonic acid (JA), gibberellic acid (GA), cytokinin (CK), and systemic acquired resistance (SAR). *P-*value ranges are marked by asterisks: ^***^ *P* < 0.01, ^**^ 0.01 < *P* < 0.05, ^*^ *P* < 0.1.

### SV treatment with 1000 Hz affects *B*. *cinerea* pathogenicity and growth in *A*. *thaliana*

**Figure 5:**
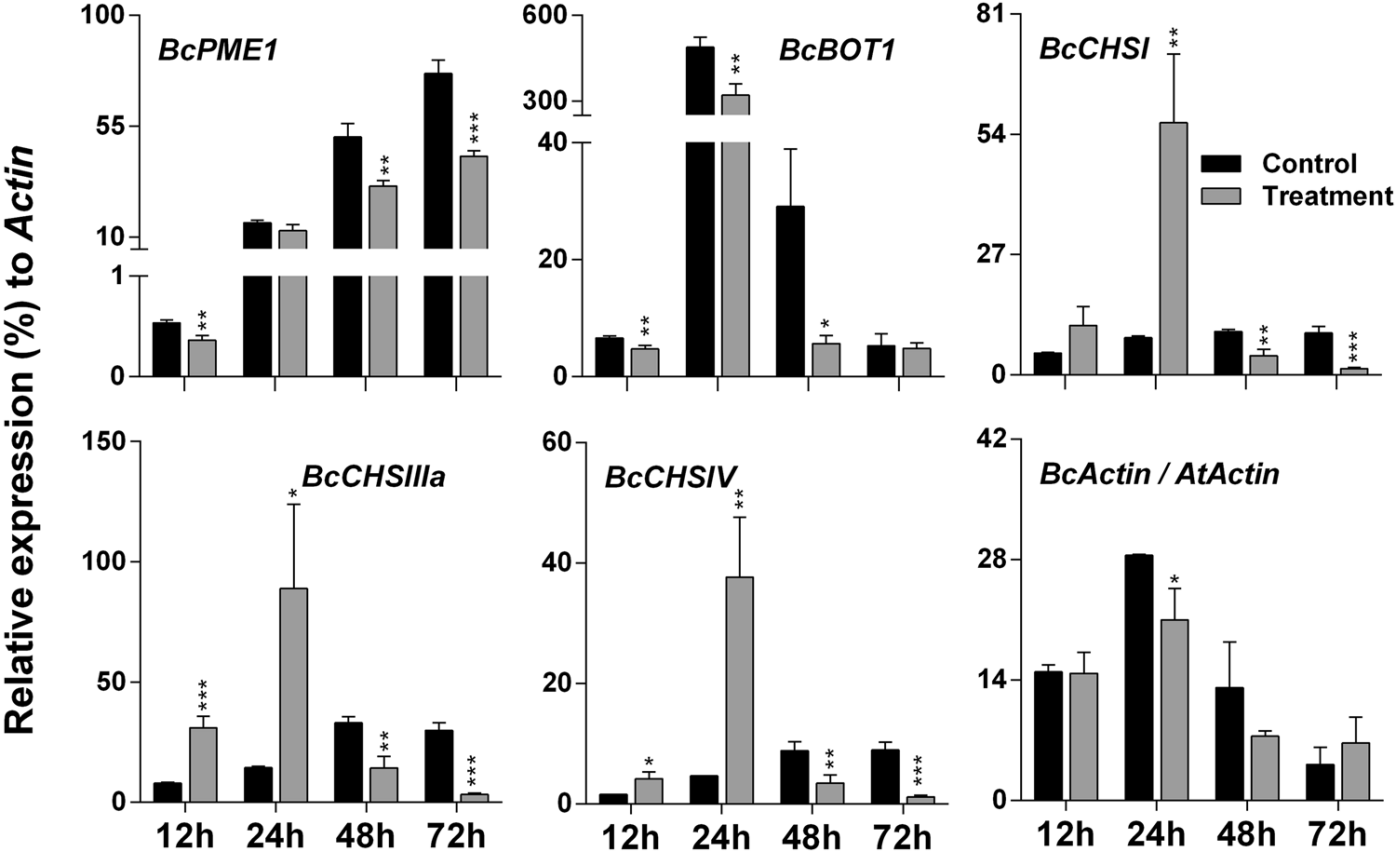
Expression patterns of fungal virulence genes after infection with *B*. *cinerea* in *A*. *thaliana* exposed to 1000 Hz sound vibration. Five representative genes for fungal growth and virulence were tested at four time points (12, 24, 48, 72 h post inoculation). Expression of each fungal gene in the Arabidopsis exposed to a 1000 Hz sound vibration (gray) was compared with control (black) after normalization with *B*. *cinerea ACTIN* (*BcACT*). Accumulation of *BcACT* transcript in the inoculated leaves was calculated relative to *A*. *thaliana ACTIN* (*AtACT*) transcript. Bars represent mean and standard error of three biological replications. *BcPME1*, encoding pectin methyltransferase; *BcBOT1*, encoding cytochrome P450 monooxygenase; *BcCHSI*, *BcCHSIIIa* and *BcCHSIV*, encoding chitin synthases. *P*-value ranges are marked by asterisks: ^***^ *P* < 0.01, ^**^ 0.01 < *P* < 0.05, ^*^ *P* < 0.1.

SV treatment increases plant disease resistance, which might be due to the alteration of plant defense mechanism, reduced pathogenicity of *B*. *cinerea*, or combination of both. To test this hypothesis, we analyzed gene expression patterns of *B*. *cinerea* using qRT-PCR in detached leaves inoculated with fungal spore (Fig. 5). The transcript level of *B*. *cinerea PME1* (*BcPME1*), encoding pectin methyltransferase that is a crucial determinant of *B*. *cinerea* virulence, was down-regulated in SV-treated plant compared to control. The transcript level for *BcBOT1*, encoding cytochrome P450 monooxygenase involved in *B*. *cinerea* phytotoxin (botrydial) synthesis, was significantly lower in SV-treated plants than control at 12, 24 and 48 hpi. Three genes encoding chitin synthases (*BcCHSI*, *BcCHSIIIa* and *BcCHSIV*) showed similar expression patterns, where they were significantly up-regulated at early time points (12 and 24 hpi) and down-regulated at later time points (48 and 72 hpi) in SV-treated plants. *B*. *cinerea ACTIN* transcript (*BcACT*) level relative to *A*. *thaliana ACTIN* (*AtACT*) was calculated to estimate the fungal growth *in planta*. *BcACT* was less in the SV-treated plants than control from 24 to 48 hpi, suggesting that the induced plant defense by SV treatment reduced fungal growth inside the host cell.

### SA-mediated response is primarily induced in 1000 Hz-treated *A*. *thaliana* during *B*. *cinerea* infection

**Figure 6:**
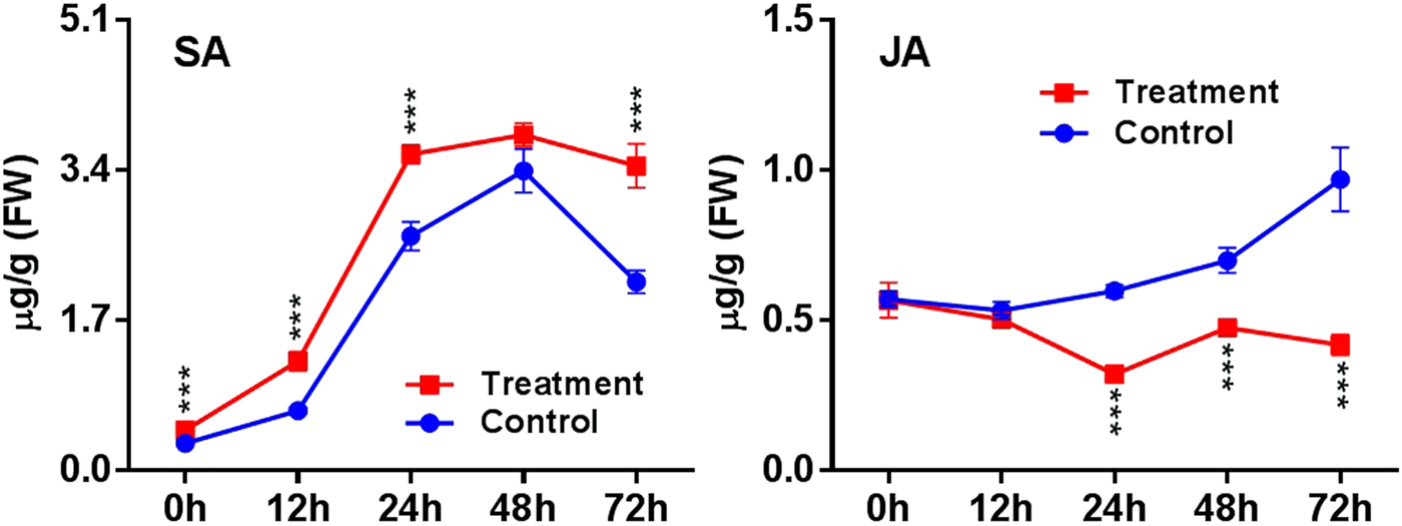
Salicylic acid (SA)- and jasmonic acid (JA)- contents after infection with *B*. *cinerea* in *A*. *thaliana* exposed to 1000 Hz sound vibration. Analysis of SA and JA contents was performed by liquid chromatography-mass spectrometry (LC-MS) and shown in a line graph, where red and blue colors indicate SV-treated and control plants respectively. Analysis was performed with three biological replications. *P*-value ranges are marked by asterisks: ^***^ *P < *0.01, ^**^ 0.01 < *P* < 0.05, ^*^ *P* < 0.1.

**Figure 7:**
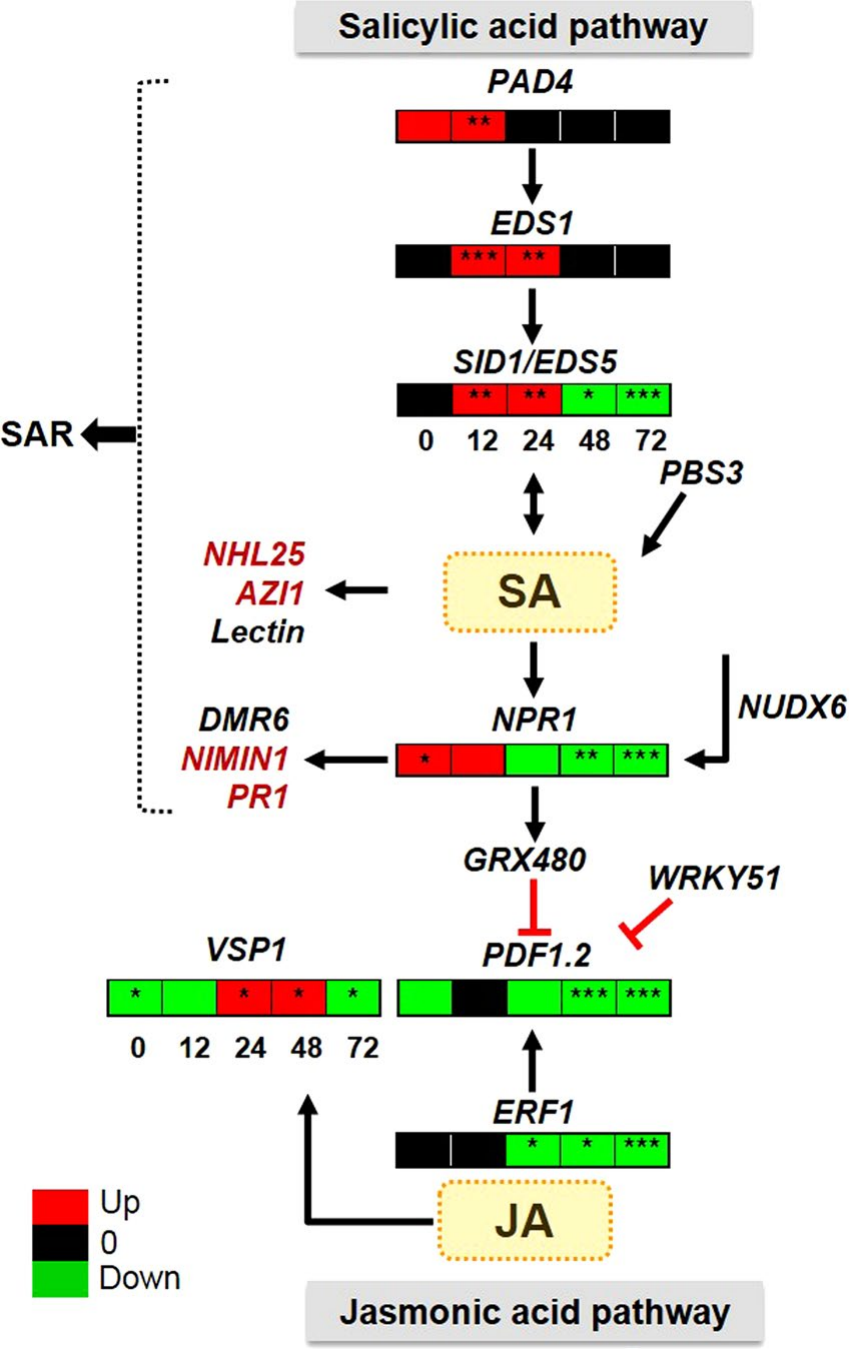
Expression pattern of the genes involved in SA- and JA-defense signaling pathways. Expression patterns are shown in colored panel where red, green, and black colors indicate transcript levels with induction, reduction and no-difference, respectively based on qRT-PCR results (Supplementary Fig. S3). The boxes within a panel indicate the expression levels of each gene in SV-treated plants compared to control during 0 to 72 h post infection (hpi). The genes marked in red color are based on the microarray results (Table 1). Pointed arrows indicate activation and blunt arrows indicate repression. All experiments were performed with three biological replications. *P*-value ranges are marked by asterisks: ^***^ *P* < 0.01, ^**^ 0.01 < *P* < 0.05, ^*^ *P* < 0.1.

Transcriptomic analyses indicate the involvement of SA –mediated signaling and subsequent resistance in the SV-treated plants. In addition, JA also plays a critical role in plant immunity and generally shows antagonism to the SA-mediated defense pathway^31^. Hence, the accumulation of SA and JA was estimated in order to ascertain their role in SV-induced fungal resistance (Fig. 6). Simultaneously expression of genes involved in SA- and JA-defense signaling was quantified (Fig. 7 and Supplementary Fig. S3). SA level in the SV-treated plants was increased compared to control plants during fungal infection from 12 to 72 hpi (Fig. 6). Moreover, increased SA concentration was observed after SV treatment relative to the untreated control at 0 h. Similarly, transcript levels of genes involved in SA-defense signaling (*PAD4*, *EDS1* and *EDS5*)^32^ were more abundant in the SV-treated plants related to control at 12 and 24 hpi (Fig. 7). In addition, *NPR1*, which is primarily involved in downstream of SA signaling, was also induced at 0 hpi in the SV-treated plants. In contrast, JA level in the SV-treated plants remained stable during the infection, while it was gradually induced in control plants (Fig. 6). Transcript levels of *PDF1*.*2*, a JA-regulated gene, and its upstream regulator *ERF1* ^31^ were reduced in the SV-treated plants relative to control at 24 to 72 hpi (Fig. 7). Unlike *PDF1*.*2*, *VSP1* was more abundant in the SV-treated plants at 24 and 48 hpi, which is one of the JA-inducible markers.

## Discussion

In this study, we demonstrated that the application of single frequency SV (1000 Hz) in Arabidopsis could induce resistance upon infection of necrotrophic fungal pathogen, *B*. *cinerea* (Fig. 1). Stronger defense response and up-regulation of defense genes in the SV-treated plants compared to that of control plants can be explained by priming^13^. In addition to the well-known chemical agents (like- SA, azelaic acid, proline, β-aminobutyric acid)^33^, mechanical stimuli has been reported to prime plants and improve the tolerance against stresses in various vegetables like bean, maize, tomato, and cucumber^15^. It has been demonstrated that SV-treatment can alter the gene expressions, hormonal levels and enzyme activities in higher plants^6, 9^, which eventually may result in increased defense metabolites and subsequent priming. The present study indicates that SV treatment could induce gene expression (*TCH4*, *LTP*, *MDAR3*, and *GRX480*) and prime Arabidopsis for potentiated gene expression (*AIG1*, *WRKY51*, *DMR6*, *MYB29*, *LECTIN*, *RLP53*, *WRKY38*, *NUDX6*, *FMO1*, *PBS3*, and *PME41*) in response to subsequent infection by *B*. *cinerea* (Fig. 4). Apparently, most of the potentiated defense genes (*NUDX6*, *WRKY51*, *WRKY38*, *PBS3*, *GRX480*, *LECTIN*, *DMR6*, *PAD4*, *EDS1*, and *EDS5*) are associated with SAR and/or SA-mediated response and significantly up-regulated at 12 and 24 hpi in the SV-treated plants compared to control plants (Figs 4 and 7). Furthermore, microarray analysis indicates that the up-regulated genes are significantly enriched with SA responsive/signaling GO term (Fig. 2a). Microarray data suggests the induction of several well-known defense-related genes (like-*AZI1*, *NHL25*, *PR1* and *NIMIN1*) in the SV-treated plants during the infection (Table 1 and Fig. 7). *AZI1*, encoding a lipid transfer protein (LTP), has a prominent role in systemic immunity priming in distal tissues^34^. *NHL25*, a SA-induced gene, is assumed to be involved in pathogenesis-related (*PR*) gene expression^35^. Up-regulation of *PR1* and *NIMIN1* was observed in the SV-treated plants which are SA-induced genes and can be controlled by NPR1^23^. *NPR1*was up-regulated by SV at 0 h (Fig. 7), which is involved in priming^13^. Overall, the transcript analyses indicate that SV might prime plants by SA-mediated response accompanied with SAR.

Recently, we have demonstrated that short-term exposure of SV treatment induces SA accumulation in Arabidopsis^9^. Increased level of SA in the SV-treated plants compared to the control plants as observed in this study further strengthens the likeliness of SV providing an impetus to the SA-mediated priming (Fig. 6). Up-regulation of *MDAR3*, a SA-induced gene^36^ and down-regulation of *GASA6*, a SA-repressed gene^37^ also corroborates with the higher concentration of SA in the SV-treated plants compared to control plants at 0 h (Figs 4 and 6). Increased concentration of SA along with stable production of JA in the SV-treated plants during infection process (Fig. 6), exhibits a non-antagonistic interaction between these two hormones. Although, the antagonistic relationship between SA and JA is often reported, synergistic interactions between these two hormones during immune response are also well-documented^31, 38, 39^. Furthermore, the genes involved in SA or JA defense signaling were co-upregulated in control as well as SV-treated plants after *Botrytis* challenge (Supplementary Fig. S3). However, the transcript levels of SA-signaling genes (*PAD4*, *EDS1*, and *EDS5*)^32^ were more abundant and those of JA- signaling genes (*ERF1* and *PDF1*.*2*)^31^ was less abundant in the SV-treated plants compared to the control plants (Fig. 7 and Supplementary Fig. S3). NPR1 has an important role in SA/JA crosstalk, such as suppression of *PDF1*.*2* by GRX480 depending on the presence of TGA factors *in planta* ^30^. High abundance of NPR1 and GRX480 transcripts at 0 and/or 12 hpi might be responsible for reduced accumulation of PDF1.2 in the SV-treated plants, related to control plants. Similarly, lesser amount of JA in SV-treated plants caused lower abundance of ERF1, compared to control plants. Although high accumulation of *VSP1* in SV-treated plants at 24 and 48 hpi, it was down-regulated at other time points (Fig. 7). VSP1 is well-known as a JA-inducible marker, which may have a role in defense against herbivores^40^. Thus, *VSP1* could be independently induced for priming against *B*. *cinerea*. Accumulation of both SA and JA was co-upregulated after *Botrytis* infection in control plants (Fig. 6). The concentration of JA was almost stable in SV-treated plants after *Botrytis* challenge. Interestingly, SA and/or its derivatives shows fungistatic effect on *Botrytis* ^41^. Therefore, elevated SA levels at 0 hpi in the SV-treated plants may hinder the successful *Botrytis* infection compared to control plants. Seemingly, the already triggered effective SA defense metabolism is enough to impede the disease progression in SV-primed plants, compared to the surging of two defense hormones together. After all, production of defense hormone requires huge energy^42^. On the other hand, co-upregulation of JA- and SA- defense signaling genes in the SV-treated plants after *Botrytis* infection might be the result of an unavoidable synergistic effect at upstream immune response. It is well-known that exogenous application of SA or its chemical analog BTH can induce resistance against *B*. *cinerea* in several plant species^43^. SA and BTH are considered as SAR inducers, which potentiate plants for priming^13^. However, exogenous SA treatment could increase resistance to *B*. *cinerea* through an apparently different mechanism than that involved in establishing the localized resistance observed in untreated plants. Overall, plants with SA- and SAR-mediated defense mechanisms can build a resistance against necrotrophic pathogens. Therefore, enhanced SA level by SV treatment might be able to protect plants from pathogens temporarily. Nonetheless, further efforts are required to establish how SA predominantly controls the defense mechanism in SV-treated plants.

Arabidopsis mutants with constitutive production of SA or defense gene expression displayed reduced growth and reproduction, while knock-out mutants blocked in induced resistance increased their growth under pathogen-free conditions^44^. However, primed plants have advantages over plants with direct induction of defense response in cost of fitness such as growth and yield^45^. In this study, we did not find any significant morphological changes after SV treatment, which might be advantageous.

It is believed that cytokinin (CK) has synergistic effect on SA signaling network and plant defense^46^. There are two types of ARRs: type A is a negative regulator of cytokinin signaling and type B is a positive regulator^46^. ARR6, a type A ARR, is an important member of CK signaling network. In our experiment, continuous up-regulation of *ARR6* (Fig. 4), indicates the possibility of CK-independent immune response, instead of SA/CK-mediated synergistic defense or balancing between downstream CK signaling and other defense hormone crosstalk.

In general, defense responses against phytopathogens are energy-consuming processes^45^. Therefore, energy metabolism needs to be reconfigured to support the increased demands for plant defense process. Reduced expression of abiotic stress related genes in the SV-treated plants at 24 hpi (Fig. 2a), might be one of the strategies to increase energy efficiency for defense metabolism. Simultaneously, the up-regulation of *CYP71A13* and *MYB29* was noted in the SV-treated plants at early stage of infection (12 and/or 24 hpi) which are involved in camalexin^47^ and glucosinolate^25^ biosynthesis, respectively (Fig. 4). In addition, *ARD3*, which is involved in methionine biosynthesis (according to gene ontology), a precursor of aliphatic glucosinolate^25^, was up-regulated in the SV-treated plants at 24 hpi. Hence, increase in defense molecules like camalexin and glucosinolates could be one of the reasons behind induced resistance in the SV-treated plants. Corroboratively, Arabidopsis rosette pre-exposed to SV caused by feeding of caterpillar exhibited effective accumulation of glucosinolate and anthocyanin which increased the defense response against herbivore^4^.

Fungal spores require sufficient time for adhesion, germination, penetration and hyphal propagation into the leaf which leads to the successful infection. *Botrytis* conidiospores require ~24 h for germination and subsequent penetration into the plant tissue^48, 49^ which causes more differential expression of genes at 24 hpi compared to 12 hpi as observed through microarray analysis (Fig. 2b). Moreover, the up-regulation of several genes, involved in cell wall biogenesis, organization, biosynthesis of wall components (like- pectin and xyloglucan) and cutin, was observed in the SV-treated plants at 24 hpi (Figs 2a and 3). This finding indicates sturdy reinforcement of the cell wall in the SV-treated plants as one of the defense strategies to prevent pathogen invasion^50^. Simultaneously, *B*. *cinerea* virulence genes were broadly down-regulated in the SV-treated plants compared to control plants after the fungal inoculation (Fig. 5). *B*. *cinerea* mutants at *BcPME1*, *BcBOT1*, *BcCHSIIIa* and *BcCHSI* loci showed reduced colonization phenotype *in planta* ^48^ which suggests their importance for successful infection to the host. Taken together, it can be stated that SV-treatment make plants more efficient to combat early infection events and reduces *Botrytis* growth temporally.

Certain perturbations with mechano-stimulation are preceded by the perception of a stimulus and signal transduction cascade. Changes in gene expression are one of the indicators of signal transduction cascade. Previously touch–induced genes (*TCH*) were identified upon exposure of various mechanical stimulation like touch and wind^51^. *TCH1*, *TCH2* and *TCH3* encode for calmodulin or calmodulin-like proteins, and *TCH4* encodes XTH^51^. Even SV treatment with audible frequencies also induced the expression of *TCH* genes in Arabidopsis^9, 52^. Thus, induction of the *TCHs* can be considered as a signature response to mechano-stimuli. It was hypothesized that soft mechanical stress (SMS) like touch could be perceived by membrane-bound mechanosensors with simultaneous triggering of damage-associated molecular patterns (DAMPs) mediated signaling, which lead to *TCH* expression and innate immune response^17^. We checked the expression level of three DAMP-marker genes (*PROPEP2*, *PROPEP3*, and *prePIP1*)^53^ to know whether SV can induce the DAMP-mediated signaling (Supplementary Fig. S4). SV-mediated up-regulation of *PROPEP2* and *PROPEP3* was observed at 0 h compared to control. Simultaneously, the up-regulation of *TCH4* was noted in the SV-treated plants compared to control at 0 h (Fig. 4). It is known that *XTHs* encode cell-wall modifying enzymes which catalyze the cleavage of xyloglucan polymers^54^. Previous studies showed that SV treatment could change cell wall fluidity, secondary structure of cell wall proteins, and ultrastructure of the membrane^6^. In response to SV, XTH could function as breaking the xyloglucan chains and making the cell wall to have more elasticity possible, which later might turn on downstream signaling cascade. However, in-depth study regarding interaction between cytoskeleton-plasma membrane-cell wall interface and SV stimulation is still required. SV causes plant leaves to vibrate which may mimic wound-mediated downstream signaling and JA accumulation^55^. Nevertheless, reduced concentration of JA in the SV-treated plants compared to control plants (Fig. 6), suggests that SV-mediated priming is distinct from wound-mediated priming at molecular level.

In summary, SV-primed Arabidopsis plants delayed the infection process against *B*. *cinerea* through SA- and SAR-mediated pathways. Previously, it has been noted that pretreatment with vibrations caused by chewing sound of caterpillar can induce Arabidopsis chemical defense against herbivore^4^. Interestingly, complex frequency profile for insect chewing and leafhopper song identifies the highest amplitude occurring between 0 - 1000 Hz^4^. Another study indicated that exposure to natural SV (like-green music) elevates the polyamines content, a chemical priming agent^33^, in Chinese cabbage^5^. Therefore, future experiments with natural SV are required to establish the ecological relevance of plant-acoustic research. Simultaneously, these findings can further apply to biotrophic pathogens where resistance is almost SA-dependent. Choosing biotrophic fungus instead of necrotrophic *B*. *cinerea*, could show stronger resistance in SV-primed plants. Comprehensive studies on SV-treated plants are warranted in future, that could open avenues in green technology for plant resistance against biotic and abiotic stresses.

## Methods

### Plant materials and SV treatment

*Arabidopsis thaliana* (Columbia-0) seeds were sown in soil mixture (Punong, Korea) and kept at 4 °C for 2 days in the dark, then transferred to growth room and grown under continuous light (~150 μmol m^−2^ s^−1^) at 23 ± 1 °C. The 14-day-old plants were exposed to SV with three separate frequencies (500, 1000 and 3000 Hz) at constant amplitude (100 dB) for daily 3 h up to 10 days in a specialized sound-proof chamber without light. The control 14-day-old plants were kept in a similar sound-proof chamber without SV exposure (daily 3 h) up to 10 days. Control and SV-treated plants were transferred back to the growth room after the daily 3 h treatment. Sound-proof chamber was customized by Korea Scientific Technique Industry as mentioned previously^56^ and single frequency SV was generated by Adobe Audition version 3.0 software. After 10 days, rosette leaves from SV-treated and control plants were harvested in liquid nitrogen, stored at −80 °C, and marked as 0 h sample. The rest of the SV-treated and control plants were used to inoculate with *Botrytis cinerea*. Inoculated plants were transferred to growth room for harvesting infected samples at various time points. A schematic diagram shown in Supplementary Fig. S1 represents the SV treatment methodology, pathogen inoculation and sample harvesting time.

### Fungal culture and disease resistance assay

*B*. *cinerea* obtained from Korea Agricultural Culture Collection (KACC) 40573 was grown on potato dextrose agar (PDA, Difco) at 23 ± 1 °C. Conidiospores were isolated according to the previous method^57^. For whole plant experiment, conidiospore suspension (3 ml) in potato dextrose broth (PDB, 5 × 10^5^ spores ml^−1^) was sprayed to both SV-treated and control plants. After spraying, the plants were grown in the growth room with transparent lid to maintain humidity. At 72 hpi, disease level was calculated in whole plant as follows: percentage of disease level (%) = (number of fully senescent leaves / total number of leaves) × 100. Rosette leaves were harvested 12, 24, 48, and 72 hpi. These samples were used for microarray, qRT-PCR, and hormone analysis.

For the experiment with detached leaves, the leaves in similar developmental stage from SV-treated and control plants were used and placed on two layers of wet Whatman filter paper in Petri dish for disease resistance assay. One drop (5 µl) of conidiospores (5 × 10^5^ spores ml^−1^ in PDB) was applied to the middle of adaxial side of leaves. The Petri dish was covered with transparent lid and kept in the growth room.

### Microarray and qRT-PCR

Total RNA was isolated from Arabidopsis rosette leaves at 0, 12 and 24 hpi using Qiagen RNeasy Plant Mini Kit according to the manufacturers’ instructions. RNA quality was examined by Agilent 2100 Bioanalyzer and quantity was determined by ND-1000 spectrophotometer (NanoDrop Technologies Inc.). RNA was labeled and hybridized to Arabidopsis Gene 1.1 ST Array according to the manufacturer’s instructions (Affymetrix). Array was scanned at GeneAtlas station and normalized based on standard Robust Multi-Average (RMA) algorithm. All microarray data have been deposited in the ArrayExpress database (https://www.ebi.ac.uk/arrayexpress/; accession no. E-MTAB-4077). For hierarchical clustering, fold change data of selected genes were processed using a commercial software package (MATLAB 7.14, The MathWorks Inc., Natick, MA, 2000). Z-score transformation, the classical method of microarray data normalization, was employed to normalize the variance in the gene expression. The hierarchical clustering of the variance in gene expression over the time was performed to reveal the co-regulated and functionally related genes by using cluster algorithms developed by Eisen *et al*.^58^. The gene expression level presented as grid of colored points are shown as red-green heatmap (Fig. 3). GO enrichment analysis of selected genes was performed through DAVID Bioinformatics Resources 6.8 (https://david.ncifcrf.gov/).

Microarray results were confirmed using qRT-PCR analysis. Total RNA (1 µg) was used to generate cDNA using cDNA synthesis kit (Promega). The qRT-PCR analysis was performed as described previously^59^ using Mx3000P QPCR system (Agilent) with SYBR Green QPCR Master Mix (LPS solution). The expression patterns of twenty Arabidopsis genes, which were selected from microarray results and additional seven genes that are involved in JA- or SA- defense signaling, were reconfirmed using qRT-PCR. The expression levels of transcripts were normalized to Arabidopsis *GAPDH* (At1g13440) encoding glyceraldehyde 3-phosphate dehydrogenase as follows: ∆C_T_ = (C_T_._GOI_)- (C_T_. _GAPDH_); where (C_T_._GOI_) and (C_T_. _GAPDH_) indicate threshold cycles for genes of interest (GOI) and *GAPDH*, respectively, for each replication^60^. Relative transcript levels of each gene were calculated with respect to *GAPDH* transcript levels (% relative expression to *GAPDH*) using 2^−∆CT^ value [2^−∆CT^ × 100] and plotted in graph^60^. Transcript levels of six *B*. *cinerea* genes (*PME1*, *BOT1*, *CHSI*, *CHSIIIa*, *CHSIV* and *ACT*) were also tested. Arabidopsis *ACTIN* (At3g18780) and *B*. *cinerea ACTIN* (AJ000335) were used to assess *B*. *cinerea* mycelial growth *in planta*. Expression level of fungal growth and virulence genes were normalized to *B*. *cinerea ACTIN* (*BcACT*) and transcript level of *BcACT* was calculated relative to *A*. *thaliana ACTIN* (*AtACT*). Primer 3 software from Biology Workbench (http://workbench.sdsc.edu/) was used to design primers. Primer details are available in Supplementary Table S3. Three independent biological replicates were used for all experiments.

### Hormonal profiling using HPLC-MS and ESI-MS/MS

Analysis of hormonal changes was performed as described previously^61^. JA (Sigma-Aldrich) and SA (Tokyo Chemical Industry Co.) were used as internal standards (IS). The stock solutions of JA and SA were prepared in 100% methanol at a concentration of 1 mg ml^−1^. The experiments were conducted using Agilent 1100 series liquid chromatography (LC) system equipped with a degasser, pump, auto sampler, and column oven. SunFire C18 column (2.1 × 10 mm, Waters) was used for chromatographic separations. The mobile phase consisted of isocratic mobile phase of 15:85 v/v 0.1% formic acid in water, 0.1% formic acid in methanol, at a flow rate of 300 µl min^−1^, column temperature of 30 °C. Sample injection was 10 µl for all experiments. MS condition was as follows: 500 °C source temperature, 5.5 kV (positive), −4.5 kV (negative) ion spray voltages, 3 collision gas (CAD), 15 curtain gas (CUR), 45 ion source gas 1 and 2, and 150 ms dwell time. Unit resolution was used for Q1 and Q3. For linearity, JA and SA standard solutions were prepared at a concentration of 0.25, 0.5, 1.0, 2.5, 5.0, 50, and 500 ng ml^−1^. Calibration curves were measured using the ratio of JA/IS and SA/IS area versus the ratio of JA/IS and SA/IS concentration. Each calibration curve was studied separately by using 1/× weighted linear regression, constructed by determining each hormone/IS peak area ratio versus each hormone/IS concentration ratio. The regression equations for the analysis were as follows: *Y* = 0.242*X* + 0.0171 (*r* = 0.9994) for JA, and *Y* = 0.704*X* + 0.115 (*r* = 0.9927) for SA using a weight factor of 1/×. The limit of detection (LOD) was determined using a signal-to-noise ratio of 3, and the limit of quantification (LOQ) was calculated using a signal-to-noise of 10. The LODs of JA and SA were 0.15 ng ml^−1^. The LOQs of JA and SA were 0.5 ng ml^−1^. Three independent biological replicates were used for hormone analysis.

### Statistical analysis

Statistical analyses with 3 independent biological replicates were performed using Student’s *t* test at *P* < 0.05. *P*-value ranges are marked by asterisks: ^***^ *P* < 0.01, ^**^ 0.01 < *P* < 0.05, ^*^ *P* < 0.1.

## Electronic supplementary material

Supplementary Figures (S1–S4) and Table S3

Supplementary Table S1

Supplementary Table S2
