# Supplementary material for: Positive regulatory role of sound vibration treatment in Arabidopsis thaliana against Botrytis cinerea infection
Source: Sci Rep. 2017 May 30;7:2527. doi: 10.1038/s41598-017-02556-9 (PMC5449397; doi:10.1038/s41598-017-02556-9)
Supplement: Supplementary file 1 — Supplementary Figures (S1–S4) and Table S3 [file 41598_2017_2556_MOESM1_ESM.pdf]

Positive regulatory role of sound vibration treatment in *Arabidopsis thaliana* against *Botrytis cinerea* infection.

Bosung Choi[1,‡], Ritesh Ghosh[1,‡], Mayank Anand Gururani[2], Gnanendra Shanmugam[1], Junhyun Jeon[1], Jonggeun Kim[1], Soo-Chul Park[3], Mi-Jeong Jeong[3], Kyung-Hwan Han[4], Dong-Won Bae[5], Hanhong Bae[1,*]

[1] Department of Biotechnology, Yeungnam University, Gyeongsan, Gyeongbuk 38541, Republic of Korea

[2] Department of Biology, College of Science, United Arab Emirates University, Al Ain 15551, United Arab Emirates

[3] National Institute of Agricultural Sciences, Rural Development Administration, Wanju, Jeollabuk 55365, Republic of Korea

[4] Department of Horticulture and Department of Forestry, Michigan State University, East Lansing, MI 48824-1222, USA

[5] Central Instrument Facility, Gyeongsang National University, Jinju, Gyeongnam 52828, Republic of Korea

[‡] **These authors contributed equally to this work:** Bosung Choi & Ritesh Ghosh

[*] **Correspondence:** Hanhong Bae (hanhongbae@ynu.ac.kr)

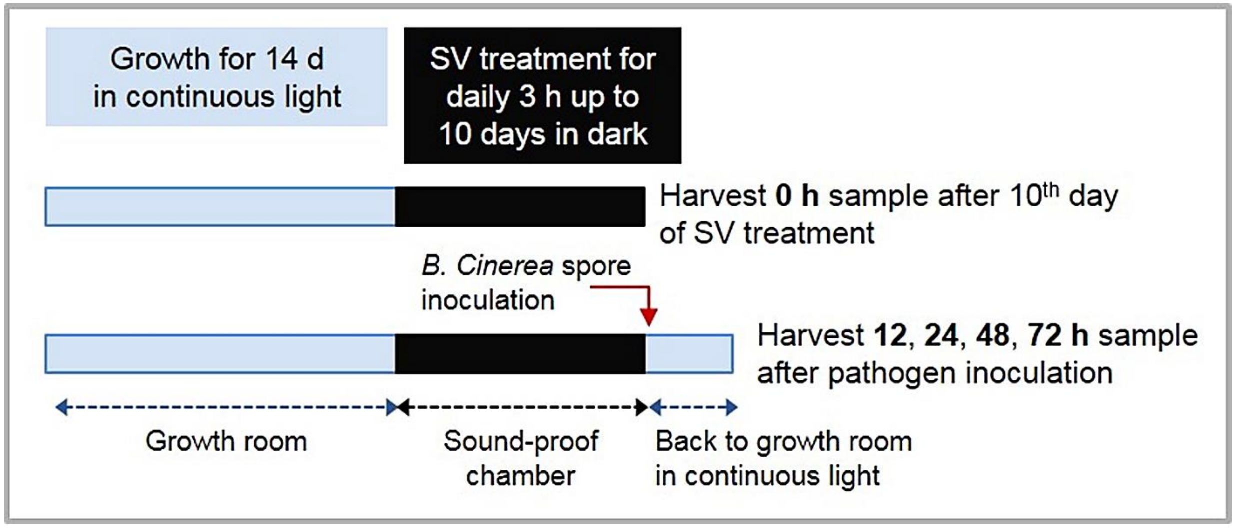

Supplementary Figure S1: Schematic representation of sound vibration (SV) treatment and sample harvest strategy. The 14-day-old plants were exposed to SV with three separate frequencies (500, 1000 and 3000 Hz) at constant amplitude (100 dB) for daily 3 h up to 10 days in a specialized sound-proof chamber without light. The control 14-day-old plants were kept in a similar sound-proof chamber without SV exposure (daily 3 h) up to 10 days. Control and SV-treated plants were transferred back to the growth room after the daily 3 h treatment. Right after the 10th day of 3 h SV treatment, 0 h sample was harvested. After harvesting 0 h sample, SV-treated and control plants were inoculated with *Botrytis cinerea* spores.

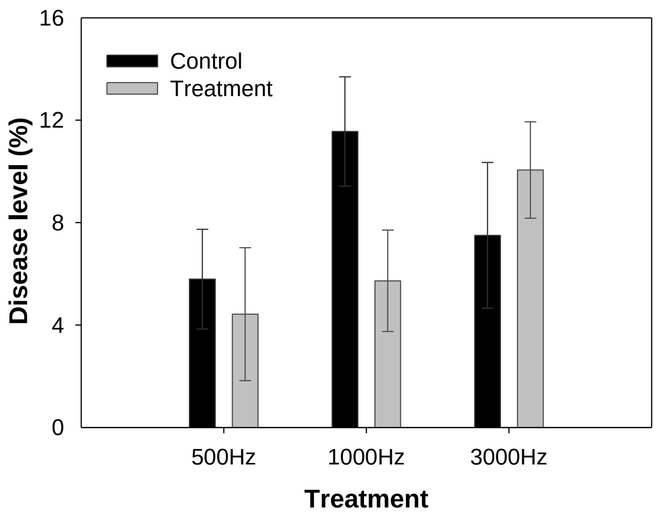

Supplementary Figure S2: Analysis of disease symptoms after infection with *Botrytis cinerea* in *Arabidopsis thaliana* exposed to 500, 1000 and 3000 Hz sound vibrations at constant amplitude (100 dB). Percentage of disease level in whole plant was calculated by counting fully senescent leaves at 72 h post inoculation (hpi) of fungal spores. Bars represent mean and standard error of four biological replications.

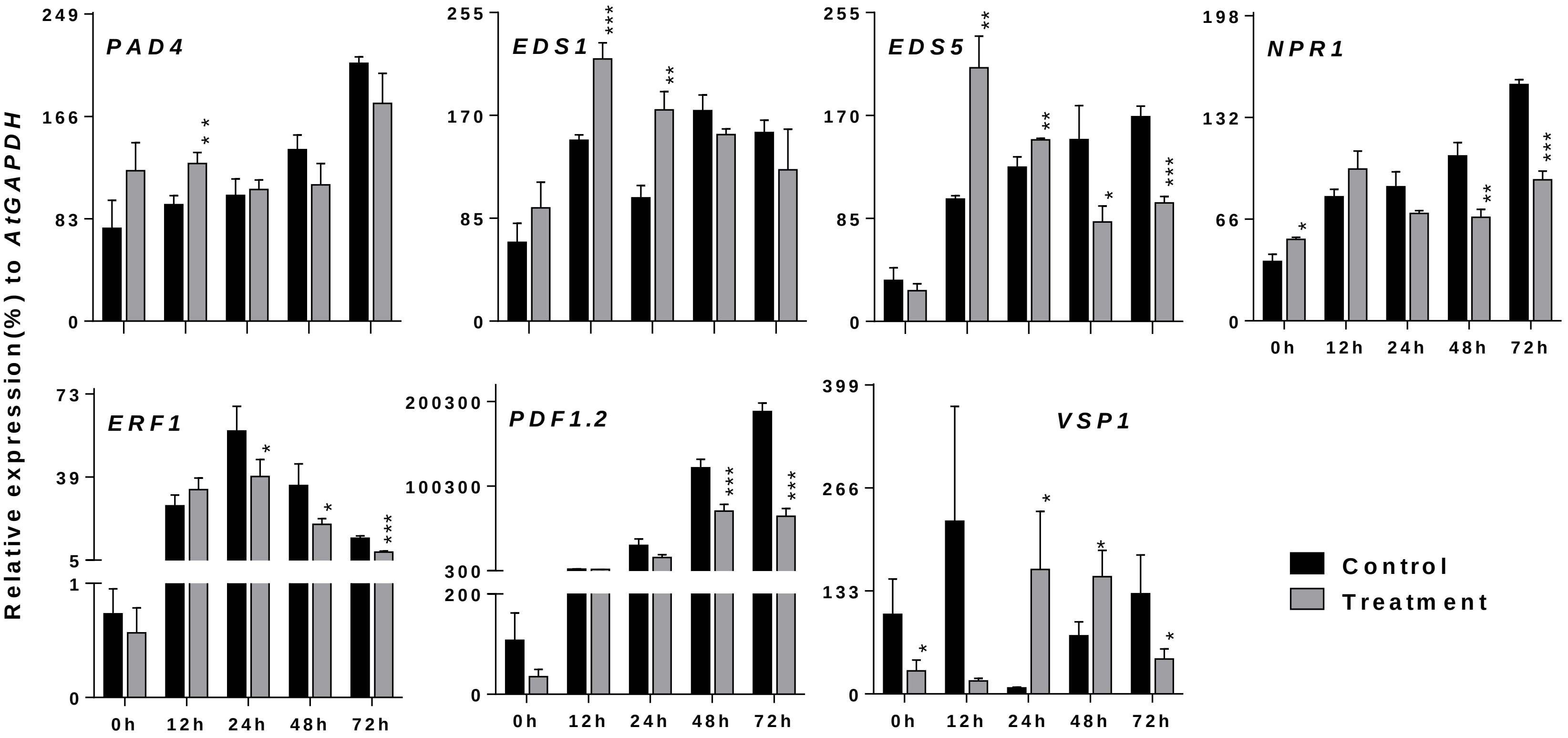

Supplementary Figure S3: Expression patterns of SA- and JA-defense signaling related genes after infection with *Botrytis cinerea* in *Arabidopsis thaliana* exposed to 1000 Hz sound vibration. The transcripts (*PAD4*, *EDS1*, *SID1/EDS5*, *NPR1*) are involved in SA-mediated signaling and *ERF1*, *PDF1.2*, *VSP1* are involved in JA-mediated signaling. Gray and black colour represents SV-treated and control plants, respectively. *GAPDH* was used for normalization. Bars represent mean and standard error of three biological replications. The time (0, 12, 24, 48 and 72 h) indicates h post inoculation (hpi). *P*-value ranges are marked by asterisks: *** $P < 0.01$, ** $0.01 < P < 0.05$, * $P < 0.1$.

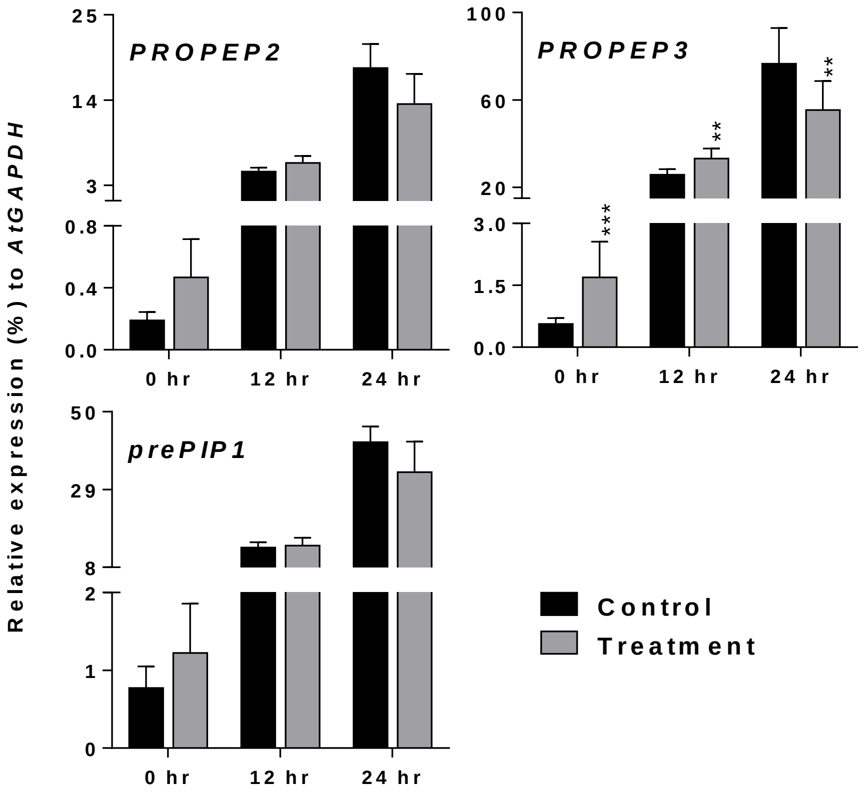

Supplementary Figure S4: Expression patterns of DAMP-responsive genes after infection with *Botrytis cinerea* in *Arabidopsis thaliana* exposed to 1000 Hz sound vibration. Three representative genes (*PROPEP2*, *PROPEP3*, and *prePIP1*) were tested at three time points (0, 12, and 24 h post inoculation). Expression of each gene in the Arabidopsis exposed to a 1000 Hz sound vibration (gray) was compared with control (black). *P*-value ranges are marked by asterisks: *** $P < 0.01$, ** $0.01 < P < 0.05$, * $P < 0.1$. DAMPs, Damage-associated molecular patterns.

Supplementary Table S3: Primer sequences used for qRT-PCR.

1. Validation of microarray - *Arabidopsis* genes

Gene name	Forward	Reverse
**DMR6 (AT5G24530)**	5′-GTGACCAGTTACAGGCATTAAG-3′	5′-AATGTTCTTGGTCCAGATTCCTACT-3′
**CYP71A13 (AT2G30770)**	5′-CGGAGAGACACTTAGATTCAGGTT-3′	5′-ATACCAATGGCTTCAGTTAGATCAG-3′
**TPS4 (AT1G61120)**	5′-GTTATCAGAAGGAACAAGAACAAGG-3′	5′-CATCTCGAAGACTTTACAACAAGAC-3′
**ARD3 (AT2G26400)**	5′-TATTGATGAAGAGATCCGTTACTGC-3′	5′-ATTATAAGCAGTCCACACTGGTCC-3′
**MDAR3 (AT3G09940)**	5′-TCTAATTGTGGGCACAGAGATAGTC-3′	5′-ATATAGTTCCATAGCCAAGGCAAG-3′
**TCH4 (AT5G57560)**	5′-GAGAATGTACTCGAGTCTTTGGAAC-3′	5′-ATCCTTTGTTGAGCTGTTGAGTC-3′
**WRKY51 (AT5G64810)**	5′-GAGGAAGTATGGCAAGAAATCTGT-3′	5′-TAAGATCGAAGAAGAGAGTGTTGGT-3′
**AIG1 (AT1G33960)**	5′-CAAGAAAGAACAAGAGGAGCTAGAA-3′	5′-ATACGTCCTTCCATCTGCTTTAACT-3′
**NUDX6 (AT2G04450)**	5′-CTCTGTGAGTTAGAACCAACCACTT-3′	5′-CTCTTTCCCAGATGAAGTCGTAGTA-3′
**MYB29 (AT5G07690)**	5′-CCATTCTATCAGTCATCTGAACACA-3′	5′-GTCAAGGAGATAATTTGACCAACC-3′
**FMO1 (AT1G19250)**	5′-CAGATTACCAAGCAAAGAGAAGATG-3′	5′-GAGTCGGTAATCTTGAGAACCATAA-3′
**WRKY38 (AT5G22570)**	5′-CCTTATCGGTTAAGCATGAAGAAG-3′	5′-ACGAGTCTGGGTTGTCAAATAAC-3′
**GRX480 (At1g28480)**	5′-AGAGGATGTTGCATGTGTCATGT-3′	5′-CTAACTCACCGGAGATATGAGTAGC-3′
**PBS3 (AT5G13320)**	5′-GTAATGGAGGAGTCGCTTGATAAT-3′	5′-ATACTGAAGAATTTGGCTACCACAC-3′
**GASA6 (AT1G74670)**	5′-AAGCTGAGTACCATCCAGAAAGTTA-3′	5′-CTTGTTGAGTCTTCCAGTTGTTGTA-3′
**LTP (AT4G12500)**	5′-AAAGAACTCAACCTCTCTTGCTCTT-3′	5′-GAAGGAGTTGGAACTGAAGGAGT-3′
**PME41 (AT4G02330)**	5′-ATCGTACATAGACGAGGTTGTTGA-3′	5′-ATAAGGTACACCACTCTGAACCATC-3′
**RLP53 (AT5G27060)**	5′-GCCTTTGAAGATAACTTGGGACT-3′	5′-AACCACTCTGGTTTATAGGAAACG-3′
**ARR6 (AT5G62920)**	5′-GAAGTACCGGTGGTAATTATGTCCT-3′	5′-GAGTGAACAGGGTAGACATTCTCTG-3′
**Lectin (AT5G03350)**	5′-AAGAGTTCAAGCTGAGTAATGGAGA-3′	5′-TTAGATTCTCTTGGCACTGTTCTG-3′

2. *Botrytis cinerea* genes

Gene name	Forward	Reverse
**BcBOT1 (AY277723)**	5′-AAGCAGCTGGAGGCATTCCCG-3′	5′-AAGCTTCTGGAGCTTGGCCGG-3′
**BcPME1 (AJ309701)**	5′-CTTTACGAGTACAAGACTGTCCGAT-3′	5′-TACAAATAACTGCTATCAACCCACC-3′
**BcchsI (X77937)**	5′-CTAGAACAATGCACGGAGTATTCA -3′	5′-ATATTCATAGATGTGTGCTGTCACG-3′
**BcchsIIIa (BC1G_04441)**	5′-CCTTGTTCTATCCATGTATCTGGTC-3′	5′-GTTGATGTAAGATGACATGAGCAAC-3′
**BcchsIV (BC1G_15275)**	5′-ACTCAAGTACCAGACTCTCTTTGGA-3′	5′-ACGACGTAGATTGTGAAAGCAATAG-3′
**BcActin (AJ000335)**	5′-GATGTCCGTAAGGATTTGTAT GGTA-3′	5′-GATCCACATCTGTTGGAAAGTAGAC-3′
**AtActin (At3g18780)**	5′-GTATGAGCAAAGAAATCACAGCACT-3′	5′-CCTTTGATCT TGAGAGCTTAGAAAC-3′

3. *Arabidopsis* JA- mediated signaling genes

Gene name	Forward	Reverse
**ERF1(AT3G23240)**	5′-ATTCTATCGGATCTTCTCCAGATTC -3′	5′-CTTACGCCTCTGTAAGACTTCTCTG -3′
**PDF1.2(AT5G44420)**	5′-CTAAGTTTGCTTCCATCATCACCC-3′	5′-TGGGACGTAACAGATACACTTGTGT-3′
**VSP1 (AT5G24780)**	5′-CCTACTACGCTAAATATGGATATGGG-3′	5′-CTTGAGATTCTCGACAGTGACTTCT-3′

4. *Arabidopsis* SA- mediated signaling genes

Gene name	Forward	Reverse
**PAD4 (AT3G52430)**	5′-GTATGAGGTGATTGATAAATGGCAG-3′	5′-GTCACCAATGTATTCGCATAACTCT-3′
**EDS1 (AT3G48090)**	5′-GAAGATGAATACAAGCCAAAGTGTC-3′	5′-GCAATATCAAGAGGCTCAACTAATC-3′
**EDS5 (AT4G39030)**	5′-CCTGGTGTCTACACACATGATAAAG-3′	5′-GTAAACCATAGCCACTTCTTGTCAC-3′
**NPR1 (AT1G64280)**	5′-GACCAGATTATGAACTGTGAGGACT-3′	5′-GACGATGAGAGAGTTTACGGTTAGA-3′

5. *Arabidopsis* DAMP-responsive genes

Gene name	Forward	Reverse
**PROPEP2 (AT5G64890)**	5′-AGCTCTCATAGCTGTCTTGAAATGT-3′	5′- GGTACACTATTAGTTTGGCCAGGA-3′
**PROPEP3 (AT5G64905)**	5′-CTCAGAAATGGAGAAGATAACGGT-3′	5′- GTAGGCTTAGTCTTGTTCTTCCCTC-3′
**prePIP1 (At4g28460)**	5′-TTGATTGTGGTTGTGATGGTGT-3′	5′- GACCTCTTGGACTTGGACCC-3′
